# Idiopathic desquamative interstitial pneumonia in a child: a case report

**DOI:** 10.1186/1756-0500-7-383

**Published:** 2014-06-22

**Authors:** Sabrina Bressieux-Degueldre, Samuel Rotman, Gaudenz Hafen, John-David Aubert, Isabelle Rochat

**Affiliations:** 1 Pediatric Pulmonology Unit, Department of Pediatrics, Centre Hospitalier Universitaire Vaudois (CHUV), Rue du Bugnon 46, 1011 Lausanne, Switzerland; 2 Institute of Pathology, Centre Hospitalier Universitaire Vaudois, Lausanne, Switzerland; 3 Division of Pulmonary Medicine, Department of Medicine, Centre Hospitalier Universitaire Vaudois, Lausanne, Switzerland

**Keywords:** Pediatric interstitial lung disease, Desquamative interstitial pneumonia, Surfactant protein dysfunction, Multiorgan involvement

## Abstract

**Background:**

Desquamative interstitial pneumonia is a rare form of interstitial lung disease in children. Respiratory symptoms appear progressively, are often subtle, and diagnosis is often delayed by a mean of 6 months after onset. High resolution chest computed tomography is the most sensitive imaging technique for demonstrating and identifying interstitial pneumonia. The typical histologic pattern of desquamative interstitial pneumonia, with prominent clustered alveolar macrophages, diffuse reactive alveolar epithelial hyperplasia and globular proteinaceous material, is diagnostic. Desquamative interstitial pneumonia in children can be idiopathic, though it is mostly related to an inborn error of surfactant metabolism.

**Case presentation:**

We present the complex clinical course and pathologic findings of a 30-months-old Mauritian and Senegalese girl with idiopathic desquamative interstitial pneumonia and multiple extrapulmonary manifestations. To our knowledge, this is the first case report of desquamative interstitial pneumonia to occur as part of a syndrome with multiple organ involvement.

**Conclusion:**

We believe that desquamative interstitial pneumonia is not always associated with mutations of the surfactant proteins, and can still be idiopathic, especially when occurring as part of a syndrome with multiple organ involvement, as described in other interstitial lung diseases.

## Background

Pediatric interstitial lung disease is a heterogeneous group of rare and diffuse lung di22333seases [1] with chronic respiratory symptoms and a negative impact on growth. It is less frequent in children than in adults, and differs considerably in its etiology and pathogenesis [2,3]. For example, desquamative interstitial pneumonia (DIP) occurs in both age groups but has different pathophysiologies. It is related to prolonged smoking in adults, whereas in children it can be idiopathic, but is mostly related to an inborn error of surfactant metabolism [4].

We describe a case of a child with idiopathic DIP diagnosed at almost 3 years of age who also presents multiple dermatologic, renal, electrolyte, neurologic, growth and developmental abnormalities since infancy. To our knowledge, this is the first case report to suggest that DIP can occur as part of a syndrome with multiple extrapulmonary manifestations, as previously described for other interstitial lung diseases [2].

## Case presentation

A 30-months-old girl was referred to our pediatric pulmonology clinic because of persistent respiratory distress and hypoxemia. Her past medical history revealed multiple cutaneous, renal, electrolyte, growth and developmental anomalies. She was born at term to a Mauritian mother and a Senegalese father, and her clinical course was marked by recurrent episodes of hyponatremia with secondary seizures, caused by a nephrogenic syndrome of inappropriate antidiuretic hormone secretion (SIADH) type C. She also had recurrent urinary tract infections, a bronchopneumonia at 3 months of age, failure to thrive, developmental delay, as well as abnormal skin pigmentation and *café-au-lait* spots. A full work-up excluded cystic fibrosis, cellular and humoral immune deficiencies, tuberous sclerosis and mitochondrial disorders. She had a normal karyotype and array-based comparative genomic hybridization (array CGH) showed no mutations in the aquaporine *AQP2* and *AVPR2* genes.

**Figure 1:**
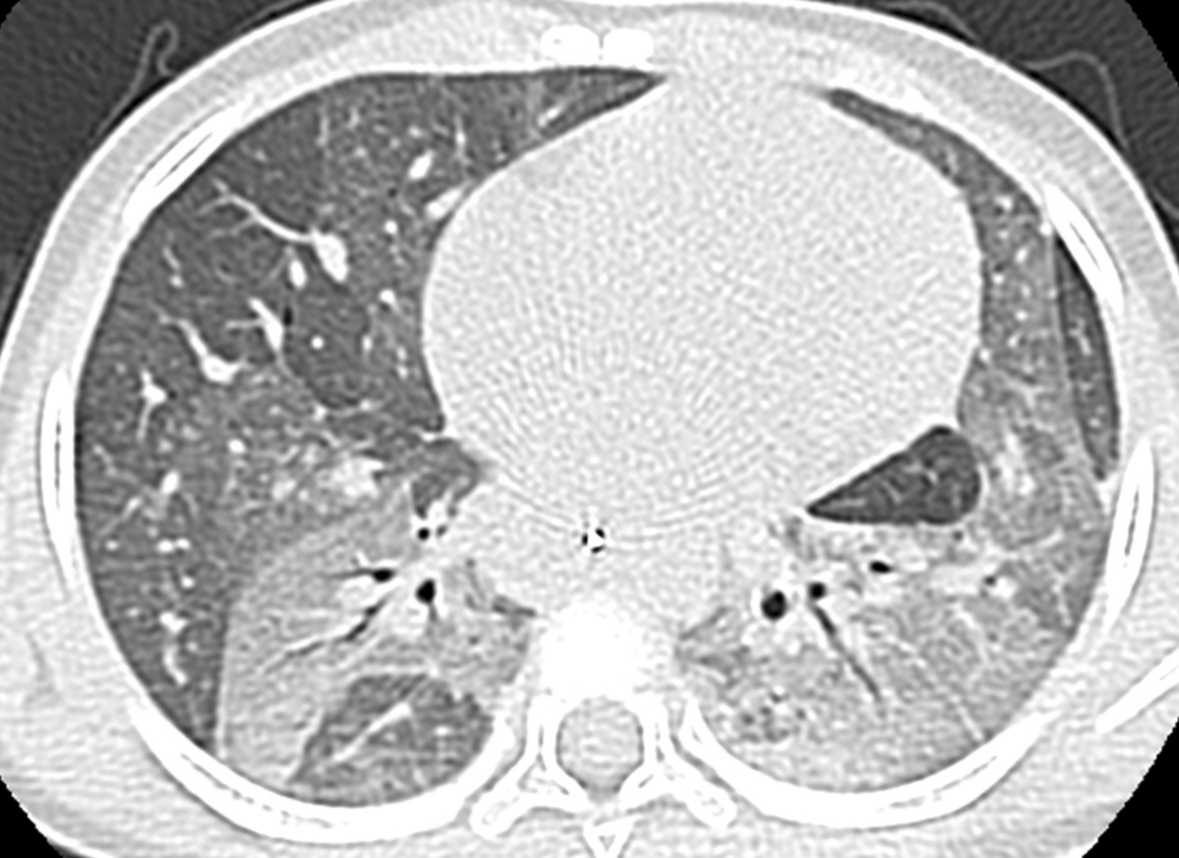
Chest computerized tomography scan showing diffuse ground-glass opacities with air-bronchograms in the upper lobes and the left lower lobe.

She was initially admitted to a regional hospital for suspected aspiration pneumonia. Despite 3 weeks of co-amoxicilline, her tachypnea, cough, respiratory distress and oxygen requirement persisted. Her physical examination revealed a respiratory rate of 42/min, a transcutaneous oxygen saturation of 92% on 1.5 L/min of supplemental oxygen by nasal cannula, retractions and fine expiratory crackles at the right apex and both bases with bronchial breath sounds on the right basal lung field. Cardiac examination was normal and there was no clubbing. Further findings included discolored fine hair, hyper- and hypopigmented macules, and *café-au-lait* spots over an extremely dry, thick and scaly dark skin.Chest radiographs consistently showed multiple right-sided confluent infiltrates with air-bronchograms and a normal heart size. In the absence of pulmonary hypertension and cardiac malformations, chronic pulmonary disease was considered. She underwent a chest high resolution computed tomography (HRCT), which showed diffuse ground-glass lung opacities (GGO) without bronchiectasis (Figure 1).

**Figure 2:**
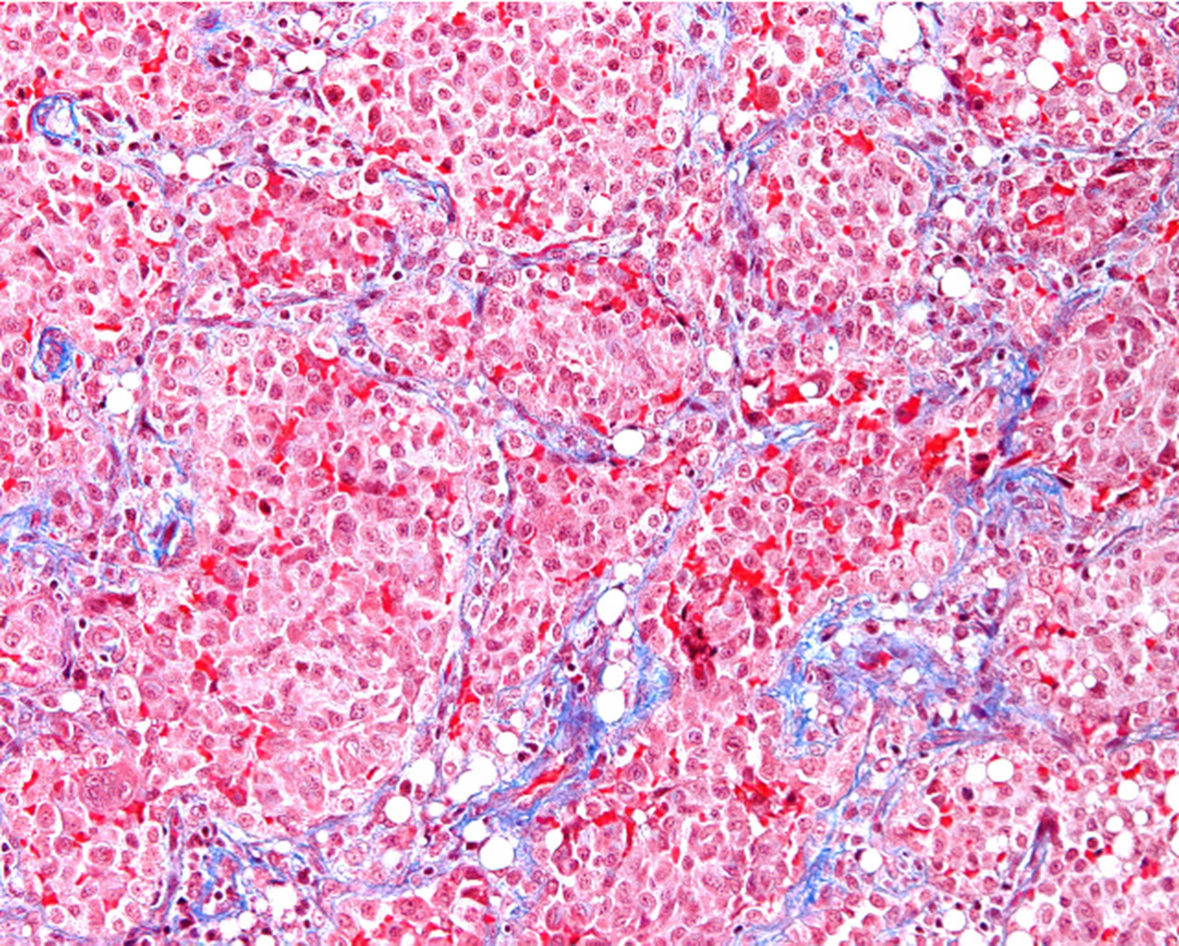
**The alveolar architecture is preserved and delimited by a fibrous thickening (blue) of alveolar walls.** Within these septae, slight mononuclear infiltrates may be seen. Alveolar spaces are filled with macrophages (red) (Trichroma, 200×).

A bronchoscopy and a lung biopsy via thoracoscopy were also performed. The bronchoalveolar lavage (BAL) showed increased cellularity with 30% lymphocytes (CD4/CD8 ratio 1.4), 45% neutrophils, and macrophagic clusters but no lipid-laden macrophages. Immunohistochemistry for histiocytosis was negative, but the presence of periodic acid-Schiff (PAS) material suggested alveolar proteinosis (PAP). However, the lung biopsy from the right upper lobe in an area of GGO did not confirm PAP. Instead, it showed macrophages completely filling the alveolar spaces, preserved pulmonary architecture, focal hyperplasia of type II pneumocytes, and fibrous thickening of alveolar septae with a monocytic inflammatory infiltrate, confirming desquamative interstitial pneumonia (DIP) (Figure 2). Both BAL and the biopsy were sterile. Since DIP is most commonly related to abnormal surfactant metabolism in children, genetic analysis for the most frequent protein mutations was performed. There were no mutations found in the deoxyribonucleic acid (DNA) sequencing of the *ABCA3* gene or in the sequence analysis of the surfactant protein C gene.

Following the diagnosis of DIP, the patient was treated with systemic corticosteroids (2mg/kg/day for 10 days then 1mg/kg/day for 1 month) and co-trimoxazole for Pneumocystis prophylaxis. Due to a poor clinical response with persistent respiratory distress and several episodes of hypoxemic malaise, hydroxychloroquine at 8mg/kg/day was added and replaced one month later by azathioprine at 2mg/kg/day due to lack of improvement.

After 2 years of treatment, the patient is still oxygen-dependent, tolerating brief daily pauses. Her dyspnea and exercise tolerance have improved slightly as she has had no further episodes of hypoxemic malaise, and she improved her walking distance on the 6-minute walk test. She hasn’t started school yet but is doing well in day care.

## Discussion

Desquamative interstitial pneumonia (DIP) is the most frequently described histologic diagnosis amongst pediatric interstitial lung diseases. Multiple causes were initially reported [5], but it is now mostly recognized as the consequence of a genetic defect in surfactant proteins.

Our patient presented with a uniquely complex medical history including renal, electrolyte, neurologic, dermatologic, growth, developmental and pulmonary anomalies. Except for nephrogenic SIADH type C, no specific disease was identified. Respiratory symptoms progressed insidiously and DIP was diagnosed 4 months after their initial appearance. As described in most forms of chronic interstitial lung disease in children, respiratory symptoms appear progressively and are often subtle, with a mean duration of at least 6 months before diagnosis [3]. Cough, tachypnea and dyspnea occur in most patients, whereas failure to thrive is more frequent in small children. The presence of these symptoms for more than 3 months should prompt specific evaluation for interstitial disease following a systematic approach [3,4].

Among ancillary tests, high resolution computed tomography of the chest is the most sensitive imaging technique for demonstrating interstitial pneumonia. Its findings may be specific for or highly suggestive of a precise diagnosis [6]. Furthermore, HRCT provides important information on the extent and severity of the disease. In our patient, the presence of ground glass opacities combined with a “crazy-paving” pattern led to a high suspicion of an inborn error of surfactant metabolism as this pattern is typically associated with PAP [6]. In addition, it helped the surgeon in selecting the appropriate lung area for biopsy.

Likewise, bronchoalveolar lavage showed PAS positive material compatible with PAP. It contributed in excluding aspiration, infection, and histiocytosis as there were no lipid laden macrophages, all cultures and viral studies were negative, and CD1a coloration was negative. The subsequent histologic examination of the lung biopsy obtained by thoracoscopy did not support PAP, but instead confirmed DIP with prominent clustered alveolar macrophages, diffuse reactive alveolar epithelial hyperplasia and globular proteinaceous material, an histologic pattern mostly related to surfactant metabolism dysfunction. However, no mutations were found in the deoxyribonucleic acid (DNA) sequencing of the *ABCA3* and *SFTPC* genes in our patient, and thyroid function was normal, excluding a *TTF1* mutation. Furthermore, electronic microscopy of the lung biopsy revealed normal lamellar bodies, making surfactant dysfunction unlikely. We therefore concluded that this was idiopathic DIP. Indeed, a small subset of patients with histologic features of genetic disorders of surfactant metabolism and normal genetic testing has been described, representing an area of active investigation [2].

There are very few publications on the co-existence of DIP and extrapulmonary manifestations. A previous report by Sheth *et al*. [7] described a patient with DIP and nephrotic syndrome due to focal segmental glomerulosclerosis with an immunologically mediated pathogenesis. In our case, immunoglobulins, serum complement levels and lymphocytic subtypes were all normal. Another report describes the development of DIP following prolonged treatment with nitrofurantoin [8]. In our case, no chronic pharmacologic treatment preceded the pulmonary manifestations. Lastly, the entity termed Reunion Island pulmonary alveolar proteinosis [9] was also excluded as it did not correspond to our patient’s ethnicity and her histologic diagnosis.

Treatment of pediatric interstitial lung disease, including DIP, is mainly supportive with aggressive nutritional intervention and prevention of infections [4]. Many children with advanced disease require supplemental oxygen therapy either at night or continuously, as was the case in our patient. Since inflammation plays an important role, corticosteroids and immunosuppressive drugs can be used but no clinical trial has proven their efficacy, and prognosis remains poor despite treatment [10]. Lung transplantation becomes an option when there is progression to end-stage disease.

In our case, several treatment options were tried, with clinical improvement achieved with low dose prednisone and azathioprinein conjunction with continuous oxygen therapy. So far, lung transplantation has not been considered.

## Conclusion

DIP is most commonly related to surfactant protein dysfunction but may be of unclear origin, especially when associated with other organ involvement. In fact, it can occur as part of a syndrome with multiple organ involvement, as described in other interstitial lung diseases. However, the relationship between chronic respiratory disease and multiorgan involvement is currently not well understood.

## Consent

Written informed consent was obtained from the patient’s parents for publication of this case report and any accompanying images. A copy of the written consent is available for review by the Editor-in-Chief of this journal.

## Abbreviations

DIP: Desquamative interstitial pneumonia; SIADH: Syndrome of inappropriate antidiuretic hormone secretion; array-CGH: array-based comparative genomic hybridization; BAL: Bronchoalveolar lavage; HCRT: High resolution computed tomography; PAS: Periodic acid-Schiff; PAP: Pulmonary alveolar proteinosis; GGO: Diffuse ground-glass lung opacities.

## Competing interests

The authors declare that they have no competing interests.

## Authors’ contributions

SBD wrote this manuscript under supervision of IR. SR performed the histopathological analysis and reviewed the pathology data. GH and JDA reviewed the clinical data. All authors read and approved the final manuscript.
